# Lifetime economic burden of prostate cancer

**DOI:** 10.1186/1472-6963-11-349

**Published:** 2011-12-28

**Authors:** Michael E Stokes, Jack Ishak, Irina Proskorovsky, Libby K Black, Yijian Huang

**Affiliations:** 1 Health Economics, United BioSource Corporation, 185 Dorval Avenue, Montreal, Quebec, H9S 5J9, Canada; 2 Global Health Outcomes, GlaxoSmithKline, 5 Moore Drive, Research Triangle Park, NC, 27709, USA; 3 Biostatistics and Bioinformatics, Emory University, Rollins School of Public Health, 1518 Clifton Road NE, Atlanta, GA, 30322, USA

**Keywords:** Cost model, lifetime costs, prostate cancer, Managed care, Medicare

## Abstract

**Background:**

Prostate cancer (PCa) is the most common cancer affecting men in the United States. The initial treatment and subsequent monitoring of PCa patients places a large burden on U.S. health care systems. The objectives of this study were to estimate the total and disease-related per-patient lifetime costs using a phase-based model of cancer care for PCa patients enrolled in Medicare.

**Methods:**

A model was developed to estimate life-time costs for patients diagnosed with PCa. Patients ≥ 65 years old and diagnosed with PCa between calendar years 1991-2002 were selected from the SEER database. Using SEER, we estimated survival times for PCa patients from diagnosis until death. The period of time patients contributed to treatment phases was determined using an algorithm designed to model the natural history of PCa. Costs were obtained from the US SEER-Medicare database and estimated during specific phases of care. Cost estimates were then combined with survival data to yield total and PCa-related life-time costs.

**Results:**

Overall, the model estimated life-time costs of $110,520 (95% CI 110,324-110,739) per patient. PCa-related costs made up approximately 31% of total costs ($34,432).

**Conclusions:**

Prostate cancer places a significant burden on U.S. health-care systems with average life-time PCa-related costs in excess of $30,000.

## Background

According to the National Cancer Institute's Surveillance Epidemiology and End Results (SEER) database, prostate cancer (PCa) prevalence in 2008 was estimated at 2,555,936 [1]. Prostate cancer is the most common cancer affecting men in the United States (U.S.) [1]. This is a result of both advances in screening following introduction of the prostate specific antigen (PSA) test as well as increases in survival attributable to more effective therapies [2]. Although incidence has leveled off in recent years, the American Cancer Society estimates that approximately 186,000 men were diagnosed with PCa in 2008 [2]. Approximately 60% of incident prostate cancer cases are diagnosed at ≥ 65 years of age [3]. The initial treatment and subsequent monitoring of these large numbers of PCa cases places a burden on U.S. health care systems. Prevalence based treatment costs for 2006 alone have been estimated at $9.6 billion [4].

Two therapies including finasteride and dutasteride have recently been studied for the prevention of PCa [5,6]. Economic burden of illness studies are important because they quantify the costs that could be avoided should these pharmacologic agents prove effective in reducing incident cancer cases. Common approaches to estimating disease-related costs often include the use of a healthcare claims database. However, databases often do not provide enough years of follow-up to estimate life-time costs. This is particularly problematic for PCa as five-year survival rates approach 100% and censored cost data become a concern [1]. Thus, there is a paucity of data concerning life-time PCa costs. This study provides estimates of total and disease-related per-patient lifetime costs using a phase-based model of cancer care among patients diagnosed with prostate cancer at ≥ 65 years of age.

## Methods

### Model Overview

We developed a phase-based model utilizing similar methodology as a prior study and applied it to PCa in order to estimate life-time total and disease-related costs for patients diagnosed with PCa [7]. Using survival data from SEER, we estimated survival times for PCa patients from diagnosis until death. The period of time that patients contributed to clinically relevant phases of treatment (initial, continuing, and terminal care) was then determined using an algorithm designed to model the natural history of PCa [7]. Finally, phase-specific monthly costs were combined with data on the number of months that patients were alive during each phase of care to yield total and PCa-related life-time costs. Analyses were conducted from a Medicare payer perspective.

### Patient Population

Survival following PCa diagnosis was estimated using data from the SEER program. At the time of analyses, SEER was collecting data on cancer incidence, survival, and prevalence from specific geographic areas representing approximately 26% of the U.S. population [8]. Male patients ≥ 65 years old with a first primary diagnosis of PCa between January 1, 1991 and December 31, 2002 were selected for inclusion from SEER using the cancer site recode variable. Patients were excluded if their cancer stage at diagnosis could not be identified or was Stage 0.

### Estimation of Survival and Length of Treatment Phases

Survival data from SEER were used to develop a statistical model designed to predict life expectancy for patients with Stage IV PCa. Time to death was calculated as the number of months between the date of index PCa diagnosis and the date of death or end of follow-up. Patients were censored if they were alive at the end of follow-up. Various parametric distributions including the exponential, Weibull, log-normal, and log-logistic were fitted to the data. Models were created with time to death as the response variable and continuous age as the independent variable. The log-normal distribution was chosen after comparing -2log-likelihood statistics for each model and comparing the median observed and predicted survival times to determine the best fitting distribution. Continuous age was included as a predictor in the models. A predictive equation was then derived from the model to estimate life expectancy for each Stage IV patient according to their age at diagnosis.

The majority of Stage I-III patients were still alive as of the last date survival data were collected in SEER. Therefore, it was not feasible to create a valid model to predict survival for these patients. For patients with no recorded date of death, we assumed survival was comparable to the U.S. general population. This was a conservative assumption based on a study by Brenner and Arndt which estimated relative survival rates for localized/regional PCa that were above 100% as compared to the general population [9]. Data from U.S. life tables based on age-specific 2004 death rates were used to estimate survival according to age and race [10].

Following estimation of survival, the time period from diagnosis until death was divided into distinct phases of care (initial, continuing, and terminal) using an approach similar to prior studies [7,11] For patients surviving ≥ 18 months, the initial phase was defined as the first 6 months following prostate cancer diagnosis, the terminal phase as the last 12 months prior to death, and the continuing care phase as the time in months between initial and terminal phases. For patients surviving < 18 months, the final 12 months of follow-up were allocated first to the terminal phase and the remaining months were defined as initial phase.

### Estimation of Monthly Treatment Phase Costs

Medicare payment data from the SEER-Medicare database was used to estimate monthly phase-specific total and disease-related medical costs [12]. Cost data were estimated from Medicare claims data spanning calendar years 1991 through 2004 for PCa cases diagnosed between January 1, 1991 and December 31, 2002 and were standardized to 2004 U.S. dollars. The main initial therapeutic options including prostatectomy, radiation, and hormone therapy were in use during the study periods over which Medicare costs were estimated. Monitoring for cancer recurrence with prostate specific antigen was also in practice.

Monthly treatment phase total and PCa-related costs that were input into the model are depicted in Table 1. PCa-related costs were estimated from the SEER-Medicare data using a sample of male patients from a random 5% sample of Medicare beneficiaries without cancer, matched to PCa cases on 5-year age groups. This non-cancer cohort was used to estimate the background medical costs unrelated to PCa. The incremental differences in the average monthly treatment phase costs between PCa patients and the cohort without cancer were defined as PCa-related costs.

**Table 1:** Mean Monthly Cost Estimates, by Stage and Treatment Phase for PCa Patients

Study Measure	Treatment Phase		
	
	Initial Care	Continuing Care	Terminal Care
Total costs			
Stage I	$2,058	$693	$3,167
Stage II	$2,333	$616	$3,044
Stage III	$2,774	$542	$3,056
Stage IV	$2,665	$790	$4,315
PCa-related costs			
Stage I	$1,615	$247	$37
Stage II	$1,890	$170	$0
Stage III	$2,331	$96	$0
Stage IV	$2,212	$344	$1,185

### Estimation of Life-time Total and PCa-related Costs

Phase-specific monthly cost estimates were combined with survival data from SEER to calculate life-time costs for patients from diagnosis until death. For each patient, the corresponding phase-specific monthly cost was multiplied by the number of months patients contributed to each treatment phase.

### Data Analyses

Baseline demographic and clinical characteristics including age, race, and geographic region were assessed descriptively by cancer stage for the PCa study cohort used to estimate survival from diagnosis until death. Life-time total and PCa-related costs were summarized by cancer stage. Confidence intervals were calculated around cost estimates using non-parametric bootstrap methods [13]. The study dataset was re-sampled with replacement to create 1,000 random samples. Total and prostate cancer-related costs were then calculated for each of the 1,000 samples. Lower and upper confidence bounds were obtained from the sample distribution of costs at the 2.5% and 97.5% quantiles, respectively. Sources of variability accounted for in the bootstrap include costs according to treatment phase and cancer stage and the number of months that patients contribute to each treatment phase. Life-time total and PCa-related costs were discounted at a 3% annual rate.

Aggregate life-time treatment costs for U.S. incident PCa cases diagnosed in 2008 and ≥ 65 years of age were calculated. By combining data from the American Cancer Society on the number of incident PCa cases (186,620) diagnosed in 2008 with the proportion of cases ≥ 65 years of age (60%) from SEER statistics, we estimated the number of incident cases 65 years of age and older (111,972) [2,3]. The number of incident cases was then multiplied by life-time treatment cost estimates in order to calculate the aggregate total and PCa-related burden of medical care among patients ≥ 65 years old.

## Results

### Patient Characteristics

Patient characteristics are shown in Table 2. One-half (51.1%) of patients were diagnosed at Stage I. Eighteen percent, 15.7%, and 14.9% were diagnosed at Stages II, III, and IV, respectively. A higher proportion of black patients were diagnosed at Stage IV (14.0%) compared to the overall proportion of black patients diagnosed in Stages I-III (10.0%). The majority of patients (57.4%) resided in the Western region at the time of PCa diagnosis. The predicted survival times from PCa diagnosis until death varied according to cancer stage (Table 2). Survival among Stage IV was shorter (Mean: 43.7 months, 3.7 years) versus Stages I-III (Mean: 178.2 months, 14.9 years). Patients diagnosed with Stage III PCa had longer mean predictive survival times compared to Stages I/II. The main driver of this result was that Stage III patients were diagnosed at younger ages (on average 2.3 years younger) versus Stages I/II (age at diagnosis: 71.3 vs. 73.6 years). The percentage of survival time Stage I-III patients spent in continuing care was higher (90.4%, 161.1 months) compared to Stage IV (66.8%, 29.2 months).

**Table 2:** Demographic and Clinical Characteristics (SEER-Medicare, incident PCa cases ≥ 65 years old diagnosed during 1991-2002)

Characteristic	Stage I		Stage II		Stage III		Stage IV		All	
N, %	71,861	51.1%	25,590	18.2%	22,103	15.7%	21,011	14.9%	140,565	100.0%
Age at study index										
Mean (SD)	73.8	(5.8)	72.9	(5.8)	71.3	(5.3)	75.30	(7.2)	73.5	(6.1)
Median	73		72		70		74		73	
Age (5-year categories), N, %:										
65-69	18,974	26.4%	8,715	34.1%	9,738	44.1%	5,424	25.8%	42,851	30.5%
70-74	22,922	31.9%	7,844	30.7%	7,378	33.4%	5,401	25.7%	43,545	31.0%
75-79	18,140	25.2%	5,486	21.4%	3,230	14.6%	4,349	20.7%	31,205	22.2%
80-84	8,235	11.5%	2,428	9.5%	1,177	5.3%	3,261	15.5%	15,101	10.7%
85+	3,590	5.0%	1,117	4.4%	580	2.6%	2,576	12.3%	7,863	5.6%
Race, N, %:										
White	58,410	81.3%	20,674	80.8%	18,327	82.9%	15,873	75.6%	113,284	80.6%
Black	7,448	10.4%	2,661	10.4%	1,839	8.3%	2,935	14.0%	14,883	10.6%
Asian	2,572	3.6%	916	3.6%	807	3.7%	861	4.1%	5,073	3.6%
Other	3,431	4.7%	1,339	5.2%	1,130	5.1%	1,342	6.3%	7,325	5.2%
Geographic region, N, %:										
Midwest	14,040	19.5%	4,531	17.7%	4,216	19.1%	4,357	20.7%	27,144	19.3%
Northeast	12,303	17.1%	3,416	13.4%	2,041	9.2%	2,426	11.6%	20,186	14.4%
South	7,024	9.8%	2,362	9.2%	1,566	7.1%	1,588	7.6%	12,540	8.9%
West	38,494	53.6%	15,281	59.7%	14,280	64.6%	12,640	60.2%	80,695	57.4%
Predicted mean survival time (SD) in months	172	(85.9)	180.1	(95.8)	196.3	(107.0)	43.7	(39.1)	158.2	(99.2)
Mean survival time (SD) in months										
Initial	5.8	(1.1)	5.8	(1.1)	5.8	(1.1)	4.4	(2.5)	5.6	(1.5)
Continuing	154.9	(85.0)	162.9	(95.1)	179.2	(106.2)	29.2	(36.5)	141.4	(98.0)
Terminal	11.8	(1.2)	11.8	(1.1)	11.8	(1.7)	10.6	(3.1)	11.6	(1.7)

N = Number of patients, SD = Standard deviation, SEER = Surveillance, Epidemiology, and End Results

### Total and PCa-related Per-Patient Life-time Costs

Average per-patient total and PCa-related life-time costs are displayed in Table 3. For all patients, the model estimated average total life-time medical-care costs of $110,520 (95% CI: 110,324-110,739). PCa-related costs ($34,432, 95% CI: 34,359-34,507) made up approximately 31% of total medical-care costs. Total medical costs ranged from $73,587-$120,085 (Stage I: $120,085, 95% CI: 119,819-120,357; Stage II: $113,616, 95% CI: 113,174-114,067; Stage III: $110,943, 95% CI: 110,525-111,412; Stage IV: $73,587, 95% CI: 73,142-74,029). Stage IV total costs ($73,587) were lower reflecting shorter periods of survival compared to patients diagnosed at lower stages. PCa-related lifetime costs were highest for Stage I ($39,182, 95% CI: 39,075-39,292), followed by Stage II ($31,915, 95% CI: 31,769-32,063), Stage IV ($30,038, 95% CI: 29,824-30,244), and Stage III ($26,078, 95% CI: 25,975-26,189). However, after adjusting PCa costs by length of follow-up, per year costs for Stage IV ($8,118) were much higher relative to Stages I ($2,740), II ($2,128), and III ($1,590), reflecting higher medical resource use intensity characteristic of distant stage patients.

**Table 3:** Mean Life-time Costs and Survival, by Cancer Stage (US$ 2004)*

Study Measure	Stage I	Stage II	Stage III	Stage IV	All Patients
Number of patients	71,861	25,590	22,103	21,011	140,565
Total costs	$120,085	$113,616	$110,943	$73,587	$110,520
PCa-related costs	$39,182	$31,915	$26,078	$30,038	$34,432
Average years of survival	14.3	15.0	16.4	3.7	13.2
Total costs per year	$8,398	$7,574	$6,765	$19,888	$8,373
PCa-related costs per year	$2,740	$2,128	$1,590	$8,118	$2,608

*Costs discounted at 3%

Aggregate life-time total costs for all incident cases ≥ 65 years and diagnosed in the U.S. during 2008 were $12.4 billion. Aggregate costs associated with PCa-related medical care totaled approximately $3.9 billion.

## Discussion

Prostate cancer places a significant burden on the U.S. Medicare system with average per-patient life-time attributable costs of approximately $34,000 (discounted at 3%). PCa-related costs represented approximately one-third of total medical care costs. Our analyses indicate that the aggregate life-time disease-related burden attributable to incident PCa cases ≥ 65 years of age in 2008 approaches $4.0B (discounted at 3%). PCa-related life-time costs were variable by cancer stage and ranged from $26,078 (Stage III) to $39,182 (Stage I). Stage IV had the poorest prognosis as the mean survival time was only 44 months compared to 172, 180, and 196 months for Stages I, II, and III, respectively.

A study conducted by Riley and colleagues examined total life-time costs of several cancers including prostate in elderly Medicare-eligible patients using the SEER-Medicare database [14]. To the best of our knowledge this is the only study conducted to date that reports life-time estimates of treatment costs. Riley *et al*. estimated costs in PCa patients ≥ 65 years of age using data from 1984 to 1990, an era pre-dating widespread use of the PSA screening test [14]. Comparison of our dataset with Riley *et al*. allows us to examine trends in survival and costs occurring over time. We report estimates of total life-time costs that are higher (Stages I, II: $118,261, Stage III: $110,943, Stage IV: $73,587) relative to Riley (Local: $98,447; Regional: $99,542; Distant: $62,634, adjusted to 2004 US$). We also estimate longer periods of survival (13.2 vs. Riley: 7.0 years). This finding is largely the result of the fact that the majority of PCa patients diagnosed in the era of widespread PSA screening that has taken place since the early 1990s are now diagnosed at earlier stages and no longer have excess mortality compared to the general population [9]. Upon adjusting total costs by year of follow-up, our estimates were actually lower ($8,373) relative to Riley *et al*. ($13,028, adjusted to 2004 US$) [14]. This finding may suggest that PCa patients can now expect to have lower morbidity related to earlier diagnosis and therefore also accrue fewer costs. Patients diagnosed at earlier stages may also be more likely to receive watchful waiting which has the effect of lowering costs relative to patients diagnosed at higher stages.

An interesting finding of our study was that Stage III PCa patients were diagnosed approximately 3.4 years younger compared to Stages I/II (age at diagnosis: 67.8 vs. 71.2 years). Prior studies examining treatment and survival outcomes using SEER registry data and data from a tumor registry in Germany have also similarly reported that Stage III patients are diagnosed at younger ages versus Stages I/II [15,16]. As part of our analysis, we assumed that survival among Stage I-III PCa patients would follow the U.S. general population based in part on 5-year relative survival statistics from SEER reporting that PCa patients diagnosed with local and regional disease is 100% [3]. Relative survival is a measure of net survival that is calculated by comparing overall survival with survival from similar individuals without cancer. We considered this to be a conservative assumption with respect to our estimation of costs since prior studies have reported relative survival for local/regional stage PCa in excess of 100% [9,16]. This finding may be explained in part by the fact that many local/regional stage tumors are diagnosed as a result of PSA screening and men who undergo this preventive measure may in fact be healthier compared to men who do not participate in PSA testing [17]. Therefore, when we estimated predicted mean survival for Stage I-III patients based on U.S. life tables, longer survival for Stage III versus Stage I/II patients was a function of their earlier mean age at PCa diagnosis as well as the assumption that survival for these patients would follow the U.S. general population. Rather than report age-adjusted survival estimates, the authors considered it important to have the survival data reflect the finding that Stage III patients are diagnosed at earlier ages relative to Stages I/II.

This study was subject to several limitations. Since PCa patients generally have a good prognosis following diagnosis and therefore long survival times, we did not have information on the date of death for the majority of patients in SEER. This was especially problematic for patients diagnosed at Stages I-III where it was assumed survival for these patients would follow the U.S. general population. This was a conservative assumption as a prior study reported relative survival rates for early stage PCa that were greater than 100% [9].

In partitioning the survival periods of patients into distinct phases of care, we used methodology similar to a study conducted by Brown and colleagues [7,11]. It should be noted that these studies were conducted in populations of colorectal, breast and lung cancer patients. However, we believe that the application of their methodology to PCa is appropriate despite the fact that many early stage patients may receive watchful waiting. In a prior study, we found that ~45% of Stage I patients received active therapy [12]. It may be argued that, for Stage I-IIa patients where a watchful waiting approach is more common, separate initial and continuing care phases may not have been warranted. However, in defining treatment phases we also looked at diagnostic procedures for cancer staging as well as outpatient visits and hospitalizations and, in our exploratory analyses, we found that more early stage PCa patients used these service types in the first 6 months following cancer diagnosis. Therefore, we considered separate initial and continuing care phases appropriate for early stage PCa. Furthermore, our analyses of healthcare costs for each treatment phase among Stage I patients showed a U-shaped pattern characteristic of patients utilizing a higher degree of medical services both in the months shortly following PCa diagnosis and in the months leading up to death compared to the months defined as continuing care.

The economic analysis was conducted from a payer perspective and includes reimbursement payments made to physicians, facilities and other healthcare professionals for the medical services that were provided to PCa patients. Other components of the economic burden of PCa including out-of-pocket spending on direct medical care as well as the indirect costs associated with reduced productivity and lost work time for both caregivers and patients were not included in this study. These cost components have been shown to represent an important proportion of the total cost burden. A study by Chang and colleagues reported monthly costs of $373, $698, and $302 for absenteeism, short-term disability, and deductibles/copayments, respectively among newly diagnosed cancer (including brain, colorectal, lung, ovarian, pancreatic, prostate, and non-Hodgkin's lymphoma) patients during the first few years following cancer diagnosis [18]. Absenteeism and short-term disability data were not available in the data sources used for the current analysis. However, this may not have contributed greatly to costs related to PCa as the population was ≥ 65 years old and not likely to have still been working. It should also be noted that Medicare cost data from calendar years 1991 through 2004 were used for this current study. During this time, Medicare did not cover most prescription drugs. Hence, prescription drug costs were not included in our estimates of direct medical costs.

It is also important to note that our survival estimates for Stage IV patients do not account for the index year of PCa diagnosis in order to adjust for improvements in prognosis among patients diagnosed in more recent years. A recent study reports that during the early 1990s until 2001, the 5-year relative survival of Stage IV patients has increased from approximately 50%-60% [19]. Therefore, this will have the effect of underestimating slightly the economic burden of PCa for recently diagnosed Stage IV patients.

Finally, it should be noted that the estimation of life-time costs is an area of research that is highly speculative [20]. Our analyses are based on the treatment experience and cost profiles of a cohort of patients diagnosed during calendar years 1991 through 2005. It is unlikely that the resource trends and costs of patients diagnosed in 2008 and more recently will exactly mirror the experience of past cohorts. For example, laproscopic and robot-assisted surgical methods are now currently more widely used. Advances have also been made in the treatment of advanced stage PCa. In 2004, it was shown that docetaxel can prolong survival in men with advanced PCa no longer responding to hormone therapy and in 2010, cabazitaxel was approved for use in advanced PCa after failure with docetaxel. As a result of these treatment advances, the PCa cost estimates reported in this study will probably understate slightly the initial and life-time treatment costs for PCa patients diagnosed today.

## Conclusions

Our analyses offer insight into the magnitude of total and disease-related lifetime costs for patients ≥ 65 years of age at PCa diagnosis in an era of improved screening and treatment (1991-2002). Long-term estimates are useful for understanding the upper limit of treatment costs that could be avoided should prevention strategies prove effective in reducing incident PCa cases. Study results indicate that the aggregate lifetime PCa-related burden for 2008 incident cases ≥ 65 years old is approximately $4.0 billion.

## Competing interests

MES, IP, and KJI are full-time employees of United BioSource Corporation (UBC). UBC received funding from GlaxoSmithKline (GSK) for the conduct of this study and the drafting of the manuscript. LKB is an employee of GSK and receives a salary from GSK. YH is a paid consultant for UBC.

## Authors' contributions

MES participated in the design and coordination of the study, analysis and interpretation of the data, and the drafting of the manuscript. IP created the study analytic file from the raw data, carried out the statistical analysis, and participated in the revision of the manuscript. LKB participated in the design of the study, analysis and interpretation of the data, and revision of the manuscript. KJI and YH participated in the design of the study and analysis and interpretation of the data. All authors read and approved the final manuscript.

## Pre-publication history

The pre-publication history for this paper can be accessed here:

http://www.biomedcentral.com/1472-6963/11/349/prepub
